# Magnetism in Nursing Education: A Qualitative Embedded Case Study of High‐Applicant Nursing Programs Amid a National Decline

**DOI:** 10.1111/jan.70295

**Published:** 2025-10-28

**Authors:** Michela Marchi, Erica Visintini, Gaia Magro, Gaia Dussi, Stefania Chiappinotto, Renzo Moreale, Chiara Moreal, Alvisa Palese

**Affiliations:** ^1^ Department of Diagnostic and Public Health University of Verona Verona Italy; ^2^ Department of Biomedicine and Prevention University of Rome Tor Vergata Rome Italy; ^3^ Department of Medicine University of Udine Udine Italy

**Keywords:** attractiveness, candidates, magnet, nursing program, nursing students, qualitative embedded case study

## Abstract

**Aims:**

To describe the factors that characterise nursing programs that continue to attract a high number of applicants even though the total number of applicants is declining.

**Design:**

A qualitative embedded case study in Italy on 2025.

**Methods:**

A purposive sample of four undergraduate nursing programs for which there were more applicants than places in the last three academic years, compared to the rest of the macro‐region, where an average ratio of 0.8 applicants/place was documented. Key informants (dean, clinical practice coordinator, nurse educators, students) from each program were involved. A semi‐structured, open‐ended interview was conducted focusing on the factors that make the identified nursing programs attractive. The recorded interviews (*n* = 19) were analysed thematically by triangulating the data. A member check was also conducted.

**Results:**

Five factors make a nursing program attractive: (1) the strategic location of the university, (2) the reputation and influence of the nursing program, (3) the structured, innovative, and open‐oriented nursing curriculum, (4) the quality of the tutorial system and (5) the program's commitment to student support and development.

**Conclusion:**

Even in times of declining enrollment and fewer applicants, certain factors can make a nursing program magnetic.

**Implications for the Profession and/or Patient Care:**

The map of emerging factors can serve as a strategy to help nursing programs attract students and improve their overall appeal.

**Impact:**

- What problem did the study address?
  ○ In some countries, there is a downward trend in applicants to the nursing profession, raising serious concerns about the growing global nursing workforce gap.
  ○ With the decline in applicants, the long‐term sustainability of nursing programs is also at risk.
  ○ No study has investigated the factors that characterise nursing degree programs, which attract even more applicants in a context of declining attractiveness.
- What were the main findings?
  ○ Five magnetic factors have emerged, one of which is external and the others internal to the nursing program.
  ○ The external factors relate to the program itself—and are embedded in the social, civic and academic environment of the host university and the city.
  ○ The internal factors relate to the strong leadership and commitment of the nursing programme to promote its quality.
- Where and on whom will the research have an impact on?
  ○ Findings may inform actions at the policy, university and individual nursing program levels.

**Reporting Method:**

COnsolidated criteria for REporting Qualitative Research Checklist.

**Patient or Public Contribution:**

Data collection and validation involved citizens (students) attending the identified nursing programs.

**Summary**

- What does this paper contribute to the wider global clinical community?
  ○ Nursing programs can seek to consider the five magnetic factors to increase candidate attractiveness.
  ○ A continuous improvement process that focuses on excellence helps attract candidates who are committed to the nursing profession.
  ○ A high‐quality nursing program enhances reputation—not only through an excellent curriculum, but also by educating well‐prepared nurses who develop into effective healthcare professionals.
  ○ Recognising strengths and developing improvement projects to increase attractiveness—even in difficult times—can increase the number of applicants and ultimately help address the nursing shortage.

## 1 Introduction

In recent years, Italy is experiencing a growing imbalance between applicants and available study places, with an overall decrease in the number of applications for Bachelor's Degree Programs in Nursing (BNSc, (FNOPI 2024)) and an increase in the number of available study places to meet the needs of the healthcare sector, which is facing a significant shortage of nurses. As a result, many universities have been unable to fill the available places (e.g., Viottini et al. 2024), raising concerns about the potential worsening of an already significant nursing shortage (Boniol et al. 2022; WHO 2024). A similar downward trend in nursing applicants has also been observed in other countries, highlighting serious concerns about the growing global nursing workforce gap (Buchan and Catton 2023). Furthermore, the decline in applicant numbers also jeopardises the long‐term sustainability of universities: If a degree program is perceived as less attractive, universities may be less inclined to invest in it, which can trigger a cycle of reduced quality, lower enrollment, and further disinvestment (Jones 2024).

While the lack of attractiveness of the nursing profession is well‐documented also in its underlying reasons (e.g., Guy et al. 2022; Mellinghoff and Blot 2024; Salminen‐Tuomaala 2023), even within this broader context of declining interest, some nursing programs continue to stand out for their ability to attract candidates. These programs show a sort of “*magnetism*,” attracting more candidates than the number of available places. The concept of “magnetism” in relation to healthcare organisations has been widely documented for Magnet Hospitals (Kramer and Schmalenberg 1988a, 1988b) and with some studies in the context of primary and secondary schools (Blank 1983; Magnet Schools of America, n.d.; Waldrip 2024). However, there are no studies that have investigated the factors (or forces) that influence this attraction (or magnetism) in the context of academic programs. Illuminating these factors may (1) open a new course of enquiry that continues the well‐established tradition of organisational research related to magnet hospitals and schools and applies it to the academic context, (2) provide insights into the current debate about the attractiveness of the nursing profession and nursing degree programs and (3) inform academic and professional strategies to promote the attractiveness of nursing degree programs.

## 2 Background

The attractiveness of the nursing profession is examined from several angles, as the propensity of young people to choose a nursing profession (e.g., Chmielewski et al. 2025) and to remain in a nursing position or within the profession itself (Savitsky et al. 2024). Attractiveness has also been investigated as “magnetism,” a concept originated in physics (Treccani, n.d.) and was applied in the 1960s in the United States (USA) to organisations whose hospitals and schools create a kind of magnetic force around them that increases their attractiveness even under difficult conditions.

Magnet Hospitals were firstly identified by the Task Force on Nursing Practice in Hospitals of the American Academy of Nursing (McClure 1983). These hospitals were considered “good places to work and good places to practice nursing” and were able to attract and retain qualified nurses at a time when there was a significant shortage of nurses (ANCC, n.d.‐a; Kramer and Schmalenberg 1988b; “Organisational Excellence,” 1984). As emerged from an interview‐based study including 41 hospitals, these “magnet” organisations had distinctive characteristics, such as visible and accessible nursing leadership with a participative management style; a high degree of autonomy of nurses and opportunities for professional development as a shared value at all levels of the hierarchy (“Organizational Excellence,” 1984). A few years later, Kramer and Schmalenberg (Kramer and Schmalenberg 1988a, 1988b) revisited the concept of magnet hospitals (“Organizational Excellence,” 1984), combining it with the eight principles of successful organisations identified by Peters and Waterman (2004). They provided an in‐depth analysis of 16 of the 41 magnet hospitals originally identified, summarising the common characteristics themed in “14 Forces of Magnetism” concerning involvement, recognition, and support of the nursing profession within the hospital organisation, leading to positive outcomes for the organisation, patients and nurses (Kramer and Schmalenberg 2005; Urden and Monarch 2002). In the 1990s, the Magnet Recognition Program, supported by the American Nurses Credentialing Center (ANCC, n.d.‐b), was established. In 2008, the ANCC Commission overseeing the Magnet Recognition Program unveiled a new vision and conceptual model that groups the forces of organisational magnetism into five key components to address the global challenges and issues facing nursing and health care: (I) transformational leadership, (II) structural empowerment, (III) exemplary professional practice, (IV) new knowledge, innovation and improvement and (V) empirical outcomes (ANCC, n.d.‐b).

In the context of education, Magnet Schools were designed as those public schools within the US established in the 1960s to contrast racial segregation and attract students through specialised curricula (Blank 1983; Magnet Schools of America, n.d.; Waldrip 2024). These were public elementary or secondary schools that offered a specialised curriculum capable of attracting a significant number of students from diverse racial backgrounds. The term “Magnet” refers here to the strategies in which these schools attract students beyond the boundaries of their home school district by utilising innovative programs linked to specific curriculum themes with the goal of desegregating schools at risk of racial isolation (Walton et al. 2018). The Magnet School model has been described by Walton and colleagues in a 2018 US Department of Education document (Walton et al. 2018). The success of this educational organisation is attributed to the integration of six key elements: (I) leadership and management, (II) communication, (III) data use, (IV) theme integration, (V) professional development and (VI) sustainability, along with the five core components, namely: diversity and equity, enrollment management, partner membership, family engagement, curriculum and instruction. The interaction between these five core components and the six pillars during the planning and implementation phase of a magnet school allows for the maximisation of services and benefits for students, families and the broader community.

Overall, even though the two research traditions—magnet hospitals and magnet schools—show no visible integration, some factors (e.g., leadership) appear to be similar. This suggests that the quality of the environment—whether in a professional or educational setting—can be attractive if certain key elements are in place.

## 3 The Study

### 3.1 Research Question and Aim

The following research question was posed: “What factors characterise BNSc that continue to attract more applicants than places, in regions where the number of applicants is declining compared to the number of places available?” The aim was to shed light on the factors that have the power to attract more applicants to some BNSc offered in regions where the number of applicants are declining.

## 4 Methods

### 4.1 Design and Theoretical Framework

A qualitative embedded case study (Kim et al. 2017; Scholz and Tietje 2002) was conducted on 2025 and here reported according to the COnsolidated criteria for REporting Qualitative research Checklist (Table S1) (Tong et al. 2007). This approach was deemed appropriate to investigate the phenomenon of BNSc attractiveness more broadly (Trinchero 2002). First, the study subunits were the BNSc identified by their capacity to attract more candidates, in a regional context where nearby nursing programs suffered from fewer applicants compared to available positions; second, the embedded case study was considered for building a generalisable theory within the replication logic of positivist case research (Eisenhardt 1989), sacrificing a degree of descriptive richness in each case; third, an explanatory approach was adopted as the research questions regarded “*how*” and “*why*” (Yin 2009) these BNSc attracted more candidates.

### 4.2 Study Setting and Participants' Involvement

The 241 Italian BNSc offered by universities across the country was first analysed according to the number of applicants for each available study place in the last three academic years. The time frame of three academic years (AYs) was established as changes in the attractiveness of nursing programs have been documented with a decreasing trend after the COVID‐19 pandemic (Brugnolli and Dimonte 2024). The research team (see authors) analysed the official data at the national level and found differences between the North, Centre and South: while about 2.17 applicants per nursing program were reported in the Southern regions (AY 2022/23), about 1.04/1.05 were documented for the Centre and North.

In the last academic year (2024/25), the North reported an average ratio of 0.82 applicants/place (−1443 candidates; Mastrillo et al. 2024); however, in three BNSc in the same macro‐region (hereafter University 1, 2 and 3), a better average ratio was recorded with more applicants than places (in all above 1), although the number of places available was increased to meet the national demand to train more nurses. In addition, one BNSc (U4) saw an increasing trend in the absolute number of applicants (from 259 in AY 2022/23 to 294 in AY 2024/25; +12%), despite an overall decrease in the number of applications in the same macro‐region (from 8310 in AY 2022/23 to 6762 in AY 2024/25; −19%) (Table S2). Therefore, the four BNSc were subjected to purposive sampling (Patton 2014), two private and two public. According to Italian law, all candidates were admitted after taking an exam; the fees for such an exam and for each year of study are shown in Table S3.

From each nursing program, there were identified key informants (Pahwa et al. 2023) as follows (a) the Dean, (b) the Coordinator of the clinical practice rotations and/or a nurse educator and (c) the Students. The participants, approached via e‐mail by the Principal Investigator (A.P.), were involved starting from the Dean, followed by the coordinator of the clinical practice rotations/educator, and then students. The latter were identified by the clinical placement coordinator among those holding representative roles or among those with significant experience in reaching the nursing program from other regions within the BNSc, as they were believed to have greater insight into the nursing program and the student experience. Moreover, to involve students who chose the BNSc rather than for geographic convenience (close to their living city), we further employed purposive sampling (Patton 2014) to engage students whose region of origin differed from that of the host university. The involvement ended when the saturation of data collected was achieved (Fusch and Ness 2015) as assessed independently and then collegially (M.M., E.V., A.P.) by researchers. All those involved agreed to participate in the study, and no refusals were recorded.

### 4.3 Data Collection

Data were collected via semi‐structured, open‐ended interviews (Kallio et al. 2016) between February and March 2025. Interview guides—developed in Italian by the research team based on existing literature (Bulgarelli et al. 2006; Kramer and Schmalenberg 1988a; Palese et al. 2010)—were tailored for: deans and coordinators of the clinical practice rotations/nurse educators; and students (Table 1). In the initial phase, data regarding the attractiveness in the last three AYs for both the specific BNSc and the north macro‐region were shown (Table S2) to each participant to share the evidence regarding the capacity to attract. A discussion was then initiated and, after familiarising themselves with the topic, the following socio‐demographic variables were collected: age, gender, institutional role and its duration, enrolment status (full‐time or part‐time), additional professional roles and their duration, while students also indicated the year of study and the distance between their place of residence and the university.

**TABLE 1:** Interview guides.

**Faculty members: Dean, coordinator of clinical practice rotations, nurse educator**
*According to the data (*Table S2 *), your nursing program seems to be magnetic…*
What elements of your institution's BNSc curriculum do you think are most attractive or appealing to potential applicants? Why do you think people apply for your BNSc? What factors most influence the attractiveness of the nursing program? Please list these
How do faculty, students or other members contribute to the attractiveness of the nursing program?
What barriers to attractiveness have you identified and what measures have you taken (or do you plan to take) to remove or mitigate these in order to attract an appropriate number of applicants?
**Students**
*According to the data (*Table S2 *), the nursing program that you are attending seems to be magnetic…*
What reasons led you to choose this BNSc over other available options?
What factors did you consider when choosing your BNSc? Please list these
What channels (e.g., university website, open days, word of mouth) did you use to find out more information when you made your nursing program choice?
Which teaching‐related features that you discovered during your studies would you now describe as particularly attractive?
What features of the clinical placement‐ that you discovered during your studies would you now find particularly attractive?
Are there any other elements of this BNSc that you discovered during your studies that you now find attractive?
Conversely, what aspects prevented you from choosing a different, closer or more accessible nursing program? Could you describe how you gathered and evaluated information about this nursing program?

Abbreviation: BNSc, Bachelor of Nursing Science course.

Interviews were audio recorded and conducted in Italian by one (M.M., female (F), registered nurse (RN), Master of Science in Nursing student (MSN)) and supervised by the second researcher (E.V., F, RN, PhD student) or the third (A.P., F, PhD, full‐time professor), either in person—in a private setting arranged with each participant—or remotely via videoconference, owing to the geographic dispersion of the selected institutions. The first two researchers (M.M., E.V.) had no prior relationship with the participants (and were not teachers of students) or limited involvement in one nursing program; the lead researcher (A.P.) had contact with some students and professors, so her participation in the data collection was limited to some interviews to avoid any influence even on the students. The dates and the locations were established jointly with the participants. Moreover, the duration of the interviews ranged from thirty minutes to more than one hour.

### 4.4 Ethical Considerations

The Institutional Review Board of the Department of Medicine at the University of Udine (Italy) approved the project (21 February 2025; protocol no. 051/2025). Participants received full information on the study aims and data‐collection procedures; there was also ensured confidentiality and they were informed of their right to withdraw at any time without justification. In particular, students were also informed that their participation (or refusal) would have no bearing on their grade or standing in the program. Written and verbally informed consent was obtained before audio‐recording; recordings were then transcribed verbatim, and all data were anonymised prior to analysis. To ensure anonymity, each interview was assigned a sequential numeric code prefixed with D (Dean, e.g., D1), CCP (Coordinator of the clinical rotations/nurse educator), S (Student), or NE (Nurse Educator).

### 4.5 Data Analysis

First, the socio‐demographic data was summarised using frequencies and percentages, means with interquartile ranges (IQR) according to the non‐normal distribution of the variables.

A qualitative thematic analysis was then conducted according to Braun and Clarke's six‐stage scheme (Braun and Clarke 2006, 2022): (a) familiarisation: M.M. transcribed all interviews verbatim, resulting in 247 pages; these were carefully read and checked for consistency (M.M., E.V., A.P.); (b) initial coding: M.M. identified preliminary codes for salient data segments; E.V. checked the coded extracts for completeness and consistency; the first draft of the codes was shared with A.P.; (c) theme development: M.M. and E.V. grouped similar codes into preliminary subthemes and then into themes, grouping all relevant codes under each, starting with subthemes and then organising them into themes; the draft was reviewed by A.P.; (d) review of subthemes and themes: through iterative discussions, M.M. and E.V. refined the theme boundaries, resolved overlaps, discarded unsupported subthemes and themes, and created a thematic map; (e) thematic definition and labelling: M.M., E.V. and A.P. agreed on concise labels and clear operational definitions for each completed theme and subthemes; (f) report writing: A.P., E.V. and M.M. selected illustrative quotes and wrote the final analytic narrative.

### 4.6 Rigour and Reflexivity

Several strategies were used to ensure rigour, theoretical credibility, transferability, reliability and confirmability (Lincoln et al. 1985). Overall rigour was ensured by jointly writing the research protocol (A.P., M.M., E.V., S.C.) and having it reviewed for consistency by an experienced educator (R.M.); we then reported the results according to the Consolidated Criteria for Reporting Qualitative Research guidelines (Tong et al. 2007) (Table S1). In addition, we formed two independent subgroups of the research team to prevent the possible influence of background and perspectives on the results: the first (A.P., M.M., E.V., S.C., R.M.) and the second (G.M., G.D., C.M.). At the beginning of the study, a joint meeting was held to share and discuss their assumptions, reasons and interests in the research topic.

To ensure credibility, three additional researchers (G.M., G.D., C.M.) independently coded a purposive subset of the interview transcripts and then held a meeting to reconcile coding inconsistencies until consensus was reached. This is how the trail code was prepared (Table S4). To ensure transferability, the criteria for sample selection, the main characteristics of the participants and the time frame of data collection were described, while an educational expert (R.M.) from a university was additionally consulted to check the appropriateness of the results. Reliability was ensured by documenting all methodological decisions, from recruitment and interview guides to coding frames and theme development. To ensure confirmability, we employed methodological triangulation by integrating the data collected from the multiple sources (deans, clinical placement coordinator and students): specifically, codes were listed according to their sources and then analysed in an integrated manner into subthemes and themes to promote a comprehensive view of the phenomenon. Once the themes were identified, a member check (Kim et al. 2017; Scholz and Tietje 2002) was conducted: a theoretical sampling method (Pahwa et al. 2023) was applied by interviewing a student living in a different region (more than 110 km) and selecting one of the participating nursing programs. Firstly, the aims and process of the study were explained, then the themes and subthemes were presented (A.P.) and discussed. All were confirmed both in their naming and in their interpretation.

The research team (see authors) encouraged reflexivity by first discussing the aims of the study and its process. Their different professional and pedagogical backgrounds were deliberately utilised to analytically enrich the study process. By documenting the discussions and taking reflective notes during the coding process, we were able to recognise how individual perspectives shaped the emerging interpretations—and adapt our analysis accordingly. The involvement of a second independent research group to review the findings (S.C., G.D., D.M., C.M., R.M.), which were subsequently discussed, further encouraged reflexivity.

## 5 Findings

### 5.1 Participants

There were 19 participants, three deans, five coordinators of clinical practice rotations, ten students and one nurse educator. Regarding the faculty members, as shown in Table 2, five were female. The average age was 45 years (range from 32 to 59 years). Their experience in the nursing program ranged from newly appointed to seven years. All worked full‐time in the academic setting, and one of them was also assigned as a clinical department head.

**TABLE 2:** Faculty members: Deans (*n* = 3), Coordinator of the clinical rotations (*n* = 5), and Nurse Educator (*n* = 1) main profile.

Characteristics	*N* = 9 (%)
Age, years, median (range)	
Deans	59 (59–59)
Coordinators	40 (32–45)
Nurse educator	36 (−)
Gender	
Female	5 (56)
Male	4 (44)
Experience in the academic role (years), median (range)	
Deans	6.5 (1.5–7)
Coordinators	3 (0.1–6)
Nurse educator	7 (−)
Employment status	
Full‐time	8 (89)
Part‐time	1 (11)
Additional roles	
No	8 (89)
Yes	1 (11)*

Abbreviation: *N*, number.

* Director of a clinical unit.

Regarding students, all were attending the nursing program as full‐time (Table 3). Overall, seven were female, and their ages ranged from 19 to 31 years (median: 23 years). Half (*n* = 5) were in their 3rd year of study; none were beyond the normal duration of study. In addition, five students had been active in various roles within the nursing program for more than one year, namely: three as class representatives, one as a member of the quality assurance committee, and two as peer mentors helping newly enrolled students enter the program. The distance between university campus and the family's home varied from 2.5 to 1600 km (Table 3). Even those who lived closest to campus indicated that they had deliberately chosen this university over other BNSc programs nearby.

**TABLE 3:** Students' main profile (*n* = 10).

Characteristics	*N* = 10 (%)
Age, years, median (range)	23 (19–31)
Gender	
Female	7 (70)
Male	3 (30)
Course year attended	
1st	4 (40)
2nd	1 (10)
3rd	5 (50)
Academic role	
No additional roles	5 (50)
Class representative	3 (30)
Peer mentor	2 (20)
Experience in the academic role, years, median (range)	
Class representative	3 (−)
Peer mentor	2 (−)
Quality Assurance Committee member	1 (−)
Student status	
Full‐time	10 (100)
Additional roles (e.g., employed)	
No	10 (100)
University‐home distance (km), median (IQR) [range]	138 (434) [2.5; 1600]
2.5–25 km	5
250–1600 km	5

Abbreviations: IQR, interquartile range; *N*, number; SD, standard deviation.

### 5.2 Magnetic Factors

**FIGURE 1:**
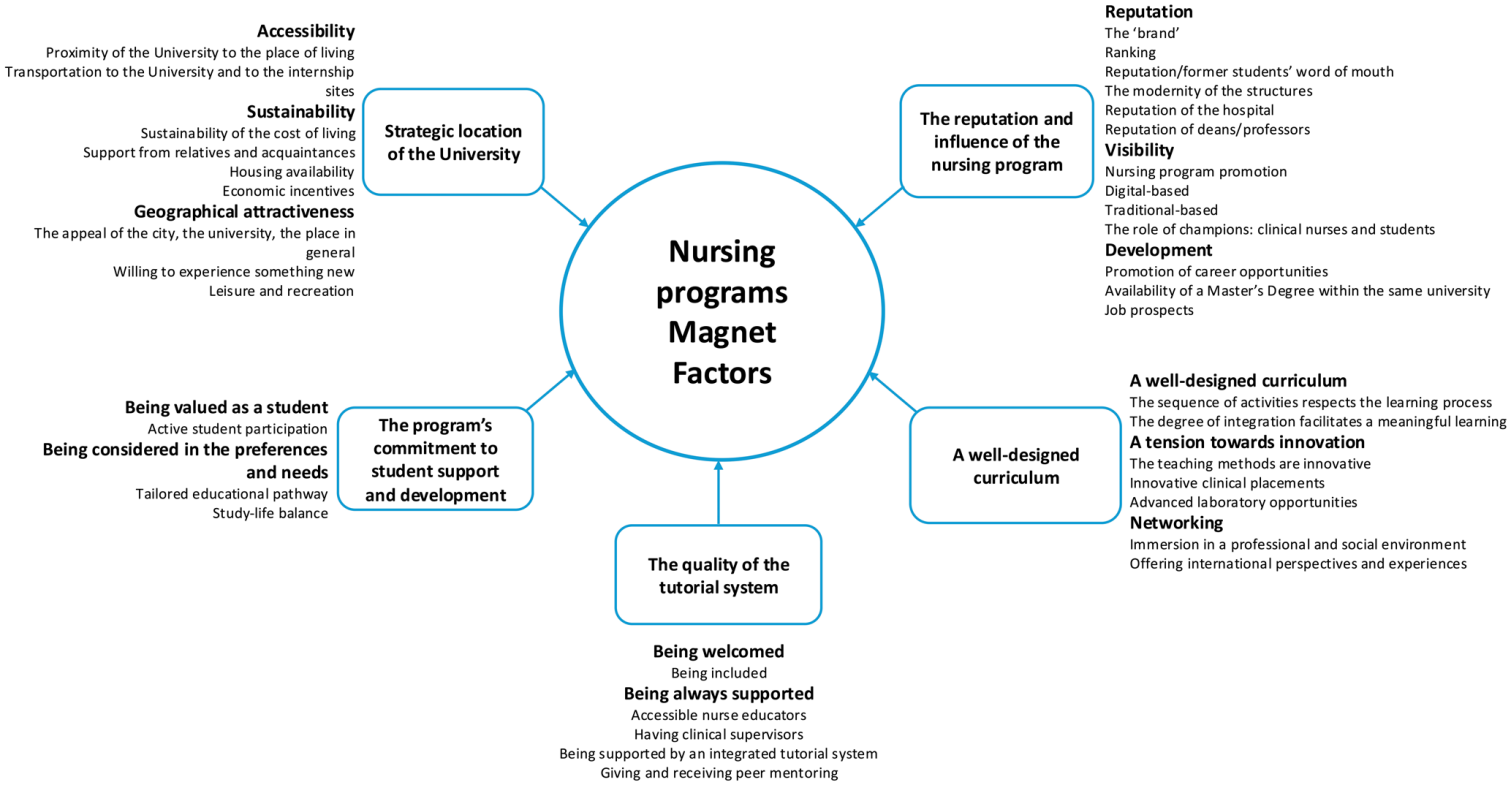
Nursing program magnet factors: Themes, subthemes and codes.

Several factors have emerged at the university, the study program, the curriculum, the tutorial system and at the student levels. As illustrated in Figure 1, these factors form a framework characterising nursing programs with more applicants than available places in a context where the number of candidates for a place is declining.

#### 5.2.1 The Strategic Location of the University

The location of the university was considered strategic in terms of (a) its accessibility from a logistical perspective, (b) its sustainability and (c) its geographical and infrastructural attractiveness.

Accessibility refers to the proximity of the university to the student's home or the availability of transportation to reach the nursing program or internship sites. The university was selected because it was close to home and easily accessible, especially by public transportation. Transportation logistics were seen as a hindrance if the university was far from the city center or poorly connected, and an advantage if efficient options—such as high‐speed rail—were available.

I immediately chose the U1 campus for purely logistical and geographical reasons (…) it was much more convenient in terms of public transportation than [another university] that was also close by, but the U1 was easier to get to because of the public transportation. (S7)

Transportation during clinical placements also has a major impact, mainly due to the geographical distribution of placements in different hospitals and the schedules that students have to follow, for example, depending on the nurse's shifts.

Sustainability includes the cost of living, support from family or acquaintances, availability of housing and access to financial aid. The university was selected based on the cost of living, which varies by city:

It's absolutely not an expensive university, and life here (…) is very sustainable. (S4)

Some students stated that a relative living close to the university influenced their decision to move away from their hometown. Others appreciated the availability of halls of residence at the university:

We have residence halls very close to campus, and students can choose to live there or find other housing nearby. (CCP3)

The availability of financial support at university or regional level, on the other hand, was important and a recognised element influencing attractiveness:

“The possibility of receiving scholarships or discounts on tuition fees (…) basically having financial advantages” (S1) and “Other attractive aspects are the benefits granted by the region, such as discounts on university fees, because our students receive a whole range of benefits.” (D3)

Geographical attractiveness includes various elements: the appeal of the university or the city itself, the student's desire to move away from home for a new experience, and the leisure and entertainment opportunities offered by the city/university. Students moved away to become independent, leave their comfort zone and focus on personal and academic growth:

First and foremost, I wanted to move away… and immerse myself in a different environment to grow academically, professionally and personally (…) Life away from home played an important role, I needed personal growth, so I decided to leave without even considering my hometown. (S6)

The fact that the university is in a small town was also perceived as important because it was considered safe. Attractiveness also depends on whether the location is central and well connected and not on the outskirts of the city. The ‘small university’ was also mentioned as students (and their parents) appreciated the familiar surroundings:

Knowing that we were in a smaller center gave them a sense of security, that we would be looked after more (…) we felt a bit more supported, not lost amongst the thousands of departments that you can find in larger universities. (CCP3)

A modern institutional infrastructure characterised by comfortable lecture halls, dedicated study spaces, a well‐stocked library, on‐campus dining options and reserved parking was cited as an important attraction, especially when all these elements are integrated into a campus that offers multidimensional experiences:

“When I first walked in, I literally got goosebumps; it says ‘This is a university for protagonists' above the entrance: They make you feel welcome, valued and like you really belong’” (S6) and “There is a large tree‐lined avenue, the football pitch, basketball and volleyball courts: There are regular tournaments organized… …there's even a piano in the main hall, so the environment itself is really pleasant to live in.” (CCP3)

The overall university environment, which can combine a good level of education with enjoyable activities, was also mentioned as a factor that plays a role in the decision to study:

They organize a lot of small parties, even sporting events, which really excite me. This gives me the feeling that university life is not just about studying, but also about enjoying and balancing everything. (S4)

#### 5.2.2 The Reputation and Influence of the Nursing Program

The nursing program was found to be strong based on (a) its ‘brand’ (reputation), (b) its visibility within and beyond the academic context and (c) its ability to provide meaningful post‐graduation development opportunities.

Reputation refers to several aspects: the ‘brand’ of the nursing program as expressed in formal rankings (e.g., national university rankings and reports) or by former students, and the modernity of the structure in which the nursing program is located; candidates also valued the reputation of the hospital where they will complete their clinical placements and that of the faculty members.

Students made decisions based on the well‐known name of the nursing program and its position in visible rankings found through search engines. Applicants routinely consult these sources and let them influence their choice, sometimes even overriding personal considerations:

When I typed ‘best hospitals, best universities, nursing, Italy’ into Google, it was always a prominent name. (S8)

Furthermore, informal word of mouth—from current students, graduates and alumni—often proves to be even more influential than official channels. Applicants inquire about the reputation of the program, clinical placements and the quality of teaching from those who are already enrolled:

More than any other official channel, talking to already enrolled students, near graduates and graduates was crucial for me. (S4)

Students also pay attention to the reputation of affiliated hospitals and prefer those that are recognised as national centers of innovation and excellence. In this regard, reputation related to the availability of a state‐of‐the‐art simulation center was also highlighted as “*a great strength to draw upon in building the image of the BNSc program*.” (CCP4)

The ability of faculty members to teach, as well as their national recognition in Italy for their efforts to advance the nursing discipline—whether as teachers, researchers, or deans—also plays an important role in shaping the reputation of the nursing program.

Visibility refers to the strategies the nursing program uses to promote itself—social media, the official website, and informational events such as ‘open days’. Social media is a welcome source of information for prospective students:

This is how students first learned about BNSc program activities and then decided to explore and participate. (CCP1)

Social accounts, managed by both the university and students, allow candidates to immerse themselves in university life, appreciate the valued role students take in the nursing program, and be in direct contact with them:

There are also student‐run sites that allow us to be even more immersed in that world … they give me a lot of reassurance; I really like what they show—simple things like the little parties they organize to make exam sessions less frustrating. (S4)

Traditional strategies such as open days with presentations about the nursing program also play an important role—especially when they involve current nursing students, whose first‐hand experiences can offer valuable insights to potential applicants and their families.

“They know how to focus on the positive aspects of the program, and they know the problems of their fellow students, so they know exactly what strings to pull to convince them” (CCP5); and “Ultimately, applicants—and often their parents—ask students questions, so they expect spontaneous answers rather than a formal presentation.” (CCP2, D2)

A part of some traditional strategies for making nursing programs visible, they offer also hands‐on workshops, “*with activities where students can get hands‐on experience to understand the different competencies that can be acquired*” (CCP4); and small group events are also often preferred over large‐scale initiatives, “…*to attract those who would not have come spontaneously*” (CCP5). In addition to these strategies, the nursing program is also committed to increasing the visibility of the nursing profession itself, which is seen as essential to promote the program's attractiveness. The involvement of both students and clinical nurses enhanced the impact of the information and orientation strategies used increasing credibility, because they “*explain who a nurse really is and what they can do*.” (CCP3)

Across all formats, the goal remains:

To also invest in what comes after graduation. (CCP1)

Effective post‐graduation strategies include the promotion of clear career pathways, the availability of an internal master's degree program and transparent employment prospects.

“The role of the nurse is poorly understood in terms of prospects, opportunities, development and career” (NE1); “There is a lack of information about educational opportunities, career goals and the potential of the profession.” (CCP4)

Therefore, efforts focusing on “*highlighting post‐graduation opportunities, both in terms of employment and further study/career*” (D3) and proposing activities aimed at “*enhancing the role of the nurse*” (CCP1) are considered important.

Applicants are attracted by the BNSc program that “*offers a master's degree within the same institution*” (NE1). Since not all universities offer a master's degree, continuity helps to retain students who are already loyal to the university. Finally, partnerships between the university and local hospitals aimed at facilitating direct recruitment of high‐performing students are highly valued:

We don't have to go through the whole recruitment process: If we are found good students, we can start working after just one interview. (S8)

#### 5.2.3 The Structured, Innovative and Open‐Oriented Nursing Curriculum

The nursing program was recognised as attractive because of its (a) a well‐designed and pedagogically coherent curriculum, (b) visible pursuit of innovation and (c) its embeddedness in a dynamic network.

The sequence of learning activities is deliberately and thoughtfully designed to support student learning. The program alternates theoretical classes, laboratory practice and clinical internships in a logical and progressive sequence. This structure allows students to engage intensively with each phase of learning without cognitive overload. Classes on specific clinical topics are routinely followed by practicums in related clinical settings, creating a cycle of applied learning. This approach is further reinforced when lab and seminar sessions coincide, allowing students to practice their skills in a protected environment before transitioning to clinical practice:

We prepare them in an initial theoretical phase before they go into the clinic, always through simulations, exercises, clinical cases and clinical reasoning. This intermediate phase reassures and prepares them (…) they feel more confident and less anxious. (CCP3)

The coherence of the parallel treatment of topics, lectures and exercises facilitates learning and helps to better memorize the concepts. (S1)

This careful coordination, when enriched with assessment planning, enhances the learning experience and contributes to a greater sense of growth and less stress:

I completed a critical care practicum in December and took the exam in January, applying the concepts I had practiced in theory and practice. (S1)

The program structure avoids overlapping activities, such as lectures and internship on the same day, which makes the experience more sustainable and effective:

If you do a morning shift and have lectures in the afternoon, you can't fully engage with the shift and approach the lectures properly. (S5)

In addition, the regularity and reliability of lectures and clinical placements—with minimal delays or absences (e.g., due to professor absences)—are highly valued by students. Examination opportunities are spread throughout the academic year, with several examination sessions per module. This flexibility allows students to manage their own study time and balance their personal lives with their academic commitments:

Having six exam dates spread throughout the year, where I can schedule my exams independently rather than having my time managed by the university… You know you have to concentrate on theory and have no other worries. (S1, S5)

This scheduling model reduces stress and prevents overlapping, allowing students to focus fully on each proposed activity—be it theoretical learning or clinical training. In addition, ongoing developments in curriculum design address the specific needs of students. New initiatives, for example, aim to support working students who often struggle with the rigid constraints of traditional curricula:

We're working on a project to ease the path of the working student who typically struggles to stay within established constraints, and that's a very sought‐after aspect. (CCP3)

Clinical placements begin early in the academic career and are designed to mirror real nursing shifts. Senior students are engaged as peer mentors in these early experiences. This model allows students to apply the theory and to experience the full trajectory of patient care, gaining insights into the responsibilities of professional nursing:

I also understand as a student what it means to do a night shift; what it means to take care of a patient for an entire shift. (S5)

Placements are offered in a variety of settings including hospitals, community care centers and institutions where nursing has a distinctive role. This diversity allows students to explore different healthcare realities and professional models and responsibilities:

Lots of contexts in which to gain experience—not just the hospital, but all other environments, including outside (…) seeing the reality of other hospitals, large and small institutions… (S1)

We have also taken into account how the needs of the population have evolved and in response we have included these types of experiences in our training package. (CCP1)

The program encourages innovation in pedagogy and clinical education. Faculty are encouraged and supported to utilise state‐of‐the‐art teaching strategies that integrate cognitive, psychomotor and relational dimensions. Small group teaching, peer education, interactive multimedia tools (e.g., interactive whiteboards), and game‐based platforms are regularly utilised.

The lecturer invited some students to give a lesson; for me that was a wonderful experience (…) I realized how challenging it really is, and it was also very, very satisfying. (S7)

In the laboratories, students benefit from realistic training experiences that simulate the complexity of nursing practice. The simulation centers, equipped with state‐of‐the‐art technology, provide a safe and controlled environment for skills development.

The program is embedded in a dynamic network that includes professional associations, hospitals and academic institutions at local and national levels; the program is also a member of international networks. These networks allow the program to embrace innovation, share best practices, and be at the forefront of progress, thus fostering growth and enriching the student experience. Students have the opportunity to complete clinical placements and volunteer work in underserved countries, broadening their professional and personal perspectives:

With internationalization in mind, a project called Honor Track was launched last year. It offers students the opportunity to do volunteer work and internships abroad, especially in countries with few resources. The proposed destinations last year were Madagascar and Tanzania. I applied, received the scholarship and was able to gain a valuable educational experience abroad. (S8)

#### 5.2.4 The Quality of the Tutorial System

An additional element that characterised the nursing program was the high‐quality tutoring system that made students feel: (a) welcome and (b) always supported. These factors include the tutorial environment that candidates experience from the beginning of enrollment and throughout their studies in the various settings, both in the classroom and in the clinical units, where they can also experience peer mentoring.

Students seek to be part of the institution and have their academic and relational needs recognised throughout their learning experience. These make students feel welcome, build trust and promote a culture of learning where mistakes are also perceived as formative:

“Everyone can express their own thoughts without fear of judgment; you are free to make mistakes and learn from those mistakes” (S7) and “There is no real distance between student and the tutors: you feel part of the nursing program.” (S1)

The tutorial system is designed to “*put the student at the center*” (CCP1) and relies on the integration of multiple roles and methods to guide and support the student throughout their journey:

That was one of the aspects that impressed me the most: the fact that you are followed and not left alone, that you are supported. (S1)

The first role the student encounters at university is the academic tutor, whose function is to liaise closely with the teaching staff, adapting learning strategies to the student's needs and building a bridge between the academic environment and the student:

One‐on‐one meetings are held once a semester between each student and their tutor to identify strengths and weaknesses. (S7)

The availability of the academic tutor ensures that students are not only welcomed but also cared for.

…students are very well looked after because there is this network of tutors who are very helpful, who are always at the university and who also respond immediately to emails, even if there are problems with lecturers or professors. (S6)

The second role faced by students is that of clinical rotations, where clinical nurses supervise students to provide ongoing support and opportunities for reflection on learning experiences, as well as formative and summative assessment. The introduction of the Dedicated Education Unit (DEU) model further strengthens the integration of theory and practice by ensuring the constant presence of the academic tutor on the ward, facilitating clinical learning and relieving clinical staff of these additional responsibilities:

It strengthens the integration between the clinical and theoretical components. (D2)

Finally, students can also meet an additional tutor: senior students are offered to become peer mentors to support younger fellow students, reduce initial anxiety and foster a collaborative community:

The opportunity to understand how to navigate a unit is a very attractive feature. (S5)

This multidimensional tutorial system in a positive environment creates a learning community where students are supported and feel at the center of the process.

#### 5.2.5 The Program's Commitment to Student Support and Development

The centrality of students within the educational pathway was also visible in these nursing programs so that (a) they felt part of the program and (b) their personal preferences and needs were considered.

Nursing programs that strongly encourage active student involvement and participation in the design and development of the curriculum demonstrate a genuine commitment to learner‐centeredness. Student involvement refers to various activities such as co‐developing new solutions for labs with faculty, preparing and conducting targeted interventions to attract more applicants, providing feedback on BNSc changes, issues or problems and evaluating the quality of clinical learning and teaching. Student feedback is specifically considered by the nursing program.

We involve them heavily, discussing with them, analyzing the data on quality, it's a team effort. (D2)

Student involvement also extends to the program's governing bodies, where class representatives and members of the Quality Committee report on problems experienced by the student community and propose changes:

Students really appreciate that their wishes are taken on board. (S2)

The needs and preferences of students are systematically collected using various strategies (surveys, interviews, one‐to‐one discussions) and then considered when making pedagogical decisions. For example, the fact that clinical placements are selected according to students' preferences is a concrete sign of the attention paid to students and emphasises the central role of students.

We are actually asked which departments we would like to do placements in… we have the opportunity to do placements in the areas we choose. (S9)

The students appreciate that their personal needs are catered for by organising the timetable flexibly to avoid overlaps between examinations and intensive work placement periods “*not overloading the placement time with exams*” (S3). Overall, they have chosen the nursing program because it offers a sustainable and balanced experience, with particular attention paid to maintaining a healthy balance between academic commitment and personal life. The curriculum is designed to ensure both viability and academic rigour, providing a comprehensive educational experience that goes beyond a purely nursing‐focused curriculum.

## 6 Discussion

### 6.1 The Study and Its Methodological Approach

To our knowledge, this is the first study to examine the “magnetic factors” of unique nursing programs, which have shown a constant ability to attract applicants over the last three years, while in the same Italian macro‐region, representing almost half of the national population (about 27 million citizens, ISTAT 2024), there has been a significant decrease in applicants (Brugnolli and Dimonte 2024). We interviewed those responsible for these programs (deans, coordinators of clinical rotations, nurse educators) and students who chose to enrol in these nursing programs. Overall, the main profile of students and that of the faculty is in line with that described in available Italian studies (e.g., Viottini et al. 2024).

From a methodological perspective, previous studies have primarily focused on the factors that attract potential students to the nursing profession. They suggest that individual and contextual motivations play an important role, such as the search for meaning and purpose through altruism and caring, for a fulfilling career, for a new direction in life, or for job security (e.g., Macdiarmid, McClunie‐Trust, et al. 2021; Macdiarmid, Turner, et al. 2021). While research has been conducted on why potential students choose nursing, the ‘*where’* and ‘*why’*, i.e., where and why a particular nursing program and the underlying reasons, have not been explored. This research gap is particularly pressing in the face of declining applicant numbers, exacerbated by the continuing decline in birth rates (e.g., D'Agostino et al. 2024). As a result, universities now face additional challenges beyond those of national healthcare systems, where the shortage of nurses appears increasingly irreversible (WHO 2022). Ensuring a consistent number of applicants each year is critical not only to the sustainability of nursing programs but also to the stability of universities, continuity of faculty, and justification of ongoing investment in nursing (Bourne 2024; Jones 2024).

### 6.2 The Magnetic Factors

Five themes have crystallised the presence of certain factors that promote the attractiveness of the nursing programs. In the available literature (e.g., Blank 1983; Kramer and Schmalenberg 1988a, 1988b; Magnet Schools of America, n.d.; Waldrip 2024), these factors have been referred to as “magnetic forces” because they create a magnetic environment that enhances attraction. Furthermore, some of the factors found have similarities to the forces that have been documented for magnetic hospitals and schools, suggesting that institutional magnetism, whether in organisations providing health services or educational services, is similar and multifaceted.

The first force refers not only to the university itself, but also to the city in which it is embedded. It is an expression of an extrinsic factor, where the power of the deans and those responsible for the nursing program to improve is limited, while the relevance of the university leadership and that of the cities is important. Ideally, nursing candidates look for institutions that are accessible, financially viable, and enjoyable, and that can provide them with a safe and multidimensional experience. Regarding the location of these universities, for example urban or rural, findings are not all in the same direction (e.g., in favour of the city), with some favouring small towns and universities, and others not. However, these elements suggest that the overall environment—safety, affordability and the opportunity to have a meaningful life experience at college—is what makes a nursing degree program attractive. With the rise of interdisciplinary approaches to learning, students may increasingly desire an integrated educational experience. As such, they may value immersion in the nursing program within a rich and stimulating context that is not solely focused on nursing. This finding seems to contrast with some of the literature that often portrays younger generations as isolated, preferring to learn at a distance (Shatto and Erwin 2017). Furthermore, it suggests the need to design nursing programs that are fully integrated into the broader academic environment—one that can promote more holistic and enriching student development, rather than focusing solely on the discipline in isolation, as has traditionally been the case with the nursing diploma.

The reputation of a nursing program, its visibility, and the opportunities it offers after graduation can be seen as key strengths that contribute to its overall attractiveness. As highlighted in the Magnet hospital model (Kramer and Schmalenberg 2005; Urden and Monarch 2002), reputation underscores the importance of both formal and informal indicators used to evaluate program performance and student satisfaction. Visibility—whether through digital technologies or word of mouth—also plays a critical role. A multidimensional strategy that integrates both traditional and digital channels, combines informative (e.g., program presentations) and experiential components (e.g., workshops), and actively involves satisfied students as ambassadors alongside practicing nurses (Cant et al. 2023) has been shown to be highly effective. In addition, nursing programs that actively promote the visibility and societal value of the nursing profession tend to increase their attractiveness. When a program is well regarded and maintains high academic standards, it attracts students who are strongly committed to their professional development—and who often choose that program over more accessible or geographically closer alternatives. This dynamic contributes to a form of applicant selection that significantly influences class composition. Programs can increasingly attract students who are highly motivated and willing to invest in their education, and who often share similar goals, expectations and attitudes.

While such an environment can promote academic excellence, it can also have the unintended consequence of decreasing diversity. Students with different educational backgrounds, learning styles or levels of preparation may find it harder to access or succeed in such selective contexts, potentially reinforcing educational inequalities (Rozendo et al. 2017). Although these strategies reinforce program identity and attractiveness—especially among younger generations seeking professional growth and clear career paths—they also pose challenges to inclusiveness. Ultimately, the effectiveness of these strategies depends on strong leadership within the nursing program, which remains a cornerstone of its reputation and long‐term success.

Overall, nursing programs are “magnetic” also when they provide a well‐structured and robust curriculum that is based on experiential learning principles, allows time for reflection, and respects the needs of students. The curriculum is attractive when it is integrated, sequential, coherent, stable (without interruption) and open to innovation and external input. In other words, when the nursing program is seriously designed and managed. The design of such a curriculum requires attention to learning theories and principles such as the integration of theory and practice, coherence between the learning phases, the sequence of activities and the avoidance of overlaps (Van Merriënboer and Sweller 2010). This approach allows time for individualised learning, ensures that assessments are spread throughout the academic year and supports effective planning of learning pathways. Overall, this suggests that candidates need clear and feasible learning pathways delivered in an orderly fashion. This should also be seen as a potential limitation when considering the complexity of preparing future generations to cope with multiple tasks and to cope with the complexity of the healthcare services that all need to be resilient.

Moreover, candidates are attracted by programs embracing innovation in their teaching methods as well as in the design of labs and clinical placements. This innovation requires a high level of digital literacy, especially considering that students are digital natives who value technologically enhanced learning tools and environments. Furthermore, networking is not only aimed at exploring advanced academic or clinical fields but also serves to promote humanitarian values. Initiatives such as clinical placements in resource‐poor countries offer students the opportunity to express their professional identity and ethical commitment through humanitarian experiences.

Another magnetic factor is identified in the tutorial system, which is characterised by its quality, its integration (academic/clinical) and its inclusiveness—where students also can take on a mentoring role for younger fellow students. This high‐quality, integrated system has long been recognised as a key component of nursing education (e.g., Zonneveld et al. 2024), confirming what has already been highlighted in the literature.

Finally, the student‐centered perspective of nursing programs has emerged as another important factor. There is widespread rhetoric about the central role of students, a concept that no university fails to explain in its official documents. However, in the nursing programs involved, this central role is tangible—it is expressed in visible actions and behaviours that students can perceive so that they truly feel a part of the program and its community, including faculty. Their needs are considered, including when it comes to leisure and well‐being. Student participation has been consistently encouraged, as has attention to their personal and academic needs (Berg and Lepp 2023). The commitment to co‐creation of processes, the value placed on feedback, and the consideration of students' individual needs reflect the recognition and the structural empowerment characteristics of Magnet hospitals (ANCC, n.d.‐a) where nurses feel recognised, centered and heard. Similarly, the consideration of students' personal lives and needs, for example in relation to clinical placements, highlights the importance of incorporating these dimensions into standards of nursing education that are overtly rigid and structured, with limited leisure activities—sometimes due to imposed rules and regulations (Ulupınar et al. 2019).

### 6.3 Strengths and Limitations

This study has both strengths and several limitations. We have taken a new line of enquiry and shifted the focus from the attractiveness of the nursing profession to the nursing programs themselves. In doing so, we drew inspiration from literature on the magnetism of other institutions (e.g., Kramer and Schmalenberg 1988a, 1988b; Waldrip 2024). While this novel research approach is promising, it needs to be substantiated by further research, especially considering that our study only included four nursing programs. The factors that emerge may reflect macro‐regional and Italian characteristics.

On the other hand, we included only four nursing programs that have attracted an above‐average number of applicants in their macro‐region over the last three years—regions where there is usually less than one applicant for every available place. However, changes over time can affect this dynamic, emphasising the need for continuous monitoring of attractiveness trends to assess the stability—and therefore robustness—of the results. In addition, the study gathered insights from individuals directly involved in the educational environment and adopting an emic perspective, i.e., from within their own cultural context (Berry 1989). The involvement of external actors (e.g., university rectors or members of the nursing board) could contribute to a more comprehensive understanding of the phenomenon. Finally, the results may not be directly transferable to other countries or educational systems due to cultural, political, economic and organisational differences. Factors such as education systems, social societal appreciation, professional prestige and economic conditions may play a different role in different contexts (e.g., OECD 2024). Further studies, including at international level, are therefore required to gain more comprehensive insights.

### 6.4 Recommendations for Further Research

This is the first study that attempts to consider the concept of magnetism in nursing education. The term ‘magnetism’ was chosen both for its physical meaning (Treccani, n.d.) related to the ability to attract and with reference to the literature that has emphasised the importance of such attractive forces in hospitals and educational settings (Walton et al. 2018). Overall, considering all limitations, this study suggests that some theories developed in one area of nursing (e.g., magnet hospitals) can be useful in advancing other areas (e.g., education) by providing insights. Sharing ideas, perspectives and research findings can therefore identify and promote new ideas that can be helpful in addressing challenges and issues in another area. Further investigation of these factors, in both high and low attraction regions and countries—where competition between nursing programs and applicants may be different—is essential for the accumulation of knowledge. Moreover, comparing the presence of these factors between non‐attractive and attractive nursing programs could confirm their value. Furthermore, transforming these factors into areas for targeted improvement initiatives and evaluating their effectiveness similar to magnet hospitals (e.g., magnet recognition programs, Urden and Monarch 2002) could provide valuable insights to help struggling nursing programs on the path to magnetism.

### 6.5 Implications for Policy and Practice

The map of emerging factors can serve as an initial strategy to support nursing programs struggling to attract candidates and to improve their overall appeal. Under this light, the findings may provide insights that can inform actions at the policy, university and individual nursing program levels. Cities and universities should form strategic partnerships to enhance the attractiveness of their institutions, considering the real needs of young people—including financial support, housing and transportation. Nursing students are a particularly unique group in this regard, as their clinical placements are highly intensive and require frequent travel to hospital sites, which leads to additional costs. At the academic level, it is essential to acknowledge the specific characteristics of nursing programs, supporting their promotion, development and innovation. At the level of individual nursing programs, efforts should focus on curriculum design and implementation, the quality of tutoring systems and the recognition of students' value and needs.

## 7 Conclusion

Even in times of declining enrollment and fewer applicants, certain factors can make a nursing program magnetic due to: (1) the strategic location of the university, (2) the reputation and influence of the nursing program, (3) the structured, innovative, and open‐oriented nursing curriculum, (4) the quality of the tutorial system and (5) the program's commitment to student support and development. The first of these factors is external to the program itself—it is embedded in the social, civic, and academic environment of the host university and city. In this context, strategies for college–city partnerships are recommended. The other factors lie within the nursing program.

A quality nursing program enhances reputation—not only through an excellent curriculum, but also by educating well‐prepared nurses who develop into effective health care professionals. In addition, increasing awareness of the program among students and practicing nurses through a variety of communication strategies that appeal to both younger and older prospective students can greatly enhance its appeal. Young people are often concerned about life after graduation. Therefore, degree programs must offer specific and concrete career prospects. A strong nursing degree program is also well designed, based on sound pedagogical principles and able to incorporate innovation while demonstrating active participation in wider academic and professional networks. It is not closed or static, but open to constant stimulation. A magnetic degree program has a robust and integrated tutorial system and takes a student‐centered approach. While some of these strengths are based on sound pedagogical principles, others are innovative and require a supportive environment to flourish.

A continuous improvement process that focuses on excellence helps attract candidates who are truly committed to the nursing profession. Benchmarking best practices, identifying strengths and developing improvement projects to promote attractiveness—even in challenging times—can increase the number of applicants and ultimately help address the nursing shortage.

## Disclosure

Permission to reproduce material from other sources: All materials drawn from previously published work (e.g., methodological frameworks, datasets) are fully cited in the reference list. No third‐party copyrighted material has been reproduced.

## Ethics Statement

The study protocol was approved by the Institutional Review Board of the Department of Medicine, University of Udine (protocol no. 051/2025, 21 February 2025).

## Consent

The authors have nothing to report.

## Conflicts of Interest

The authors declare no conflicts of interest.

## Supporting information


**Table S1:** COnsolidated criteria for REporting Qualitative Research Checklist.

**Table S2:** Applicants and places in the last three academic years in the North (detailed for each nursing program) in the Centre (on average) and in the South (on average) of Italy.

**Table S3:** University cost of entrance examination and fees.

**Table S4:** The trail code.

## Data Availability

The datasets generated during the current study are available from the corresponding author on reasonable request.
