# Supplementary material for: Magnetism in Nursing Education: A Qualitative Embedded Case Study of High‐Applicant Nursing Programs Amid a National Decline
Source: J Adv Nurs. 2025 Oct 28;82(6):6586–601. doi: 10.1111/jan.70295 (PMC13176678; doi:10.1111/jan.70295)
Supplement: Supplementary file 2 — Table S2: Applicants and places in the last three academic years in the North (detailed for each nursing program) in the Centre (on average) and in the South (on average) of Italy. [file JAN-82-6586-s003.docx]

**Supplementary Table 2.** Applicants and places in the last three academic years in the North (detailed for each nursing program) in the Centre (on average) and in the South (on average) of Italy

|  |  | | | | **AY 2022-2023** | | | | **AY 2023-2024** | | | | **AY 2024-2025** | | | |
| --- | --- | --- | --- | --- | --- | --- | --- | --- | --- | --- | --- | --- | --- | --- | --- | --- |
|  |  |  |  |  | **Applicants** | **Places** | **A-P** | **A/P** | **Applicants** | **Places** | **A-P** | **A/P** | **Applicants** | **Places** | **A-P** | **A/P** |
| **NORTH OF ITALY** | | | | | | | | | | | | | | | | |
| **Bachelors in Nursing Science** | | | | | | | | | | | | | | | | |
| **U1** |  |  |  |  | 312 | 205 | 107 | **1.52** | 325 | 215 | 110 | **1.51** | 310 | 235 | 75 | **1.32** |
| **U2** |  |  |  |  | 243 | 140 | 103 | **1.74** | 230 | 140 | 90 | **1.64** | 254 | 140 | 114 | **1.81** |
| **U3** |  |  |  |  | 240 | 185 | 55 | **1.30** | 241 | 180 | 61 | **1.34** | 210 | 200 | 10 | **1.05** |
| **U4** |  |  |  |  | 259 | 324 | -65 | **0.80** | 309 | 341 | -32 | **0.91** | 294 | 280 | 14 | **1.05** |
| [other 14 Universities omitted] | | | |  |  |  |  |  |  |  |  |  |  |  |  |  |
| **NORTH OF ITALY, overall** | | | | | | | | | | | | | | | | |
|  |  |  |  |  | **8,310** | **7,943** | **+367** | **1.05** | **7,269** | **8,167** | **-898** | **0.89** | **6,762** | **8,205** | **-1,443** | **0.82** |
| **CENTER OF ITALY, overall** | | | | | | | | | | | | | | | | |
|  |  |  |  |  | **6,991** | **6,711** | **+280** | **1.04** | **5,923** | **6,734** | **-811** | **0.88** | **5,434** | **6,790** | **-1,356** | **0.80** |
| **SOUTH OF ITALY, overall** | | | | | | | | | | | | | | | | |
|  |  |  |  |  | **10,238** | **4,721** | **+5,517** | **2.17** | **9,765** | **5,158** | **+4,607** | **1.89** | **8,982** | **5,539** | **3,443** | **1.62** |

Legend: A, Applicants; P, Places; U1, 2, 3… University 1, Bachelor of Nursing Science Course n. 1, anonymised.

Adapted from: Mastrillo, A., Bevacqua, L., & Cenerelli, E. (2024). CORSI DI LAUREA DELLE PROFESSIONI SANITARIE Dati sull’accesso ai Corsi e programmazione dei posti nell’ A.A. 2024-25 Quotidiano Sanità [Degree Programs in Healthcare Professions
Data on Admissions and Seat Allocations for the 2024–25 Academic Year – Quotidiano Sanità]. Retrieved 19/05/2025 from <https://www.quotidianosanita.it/allegati/allegato1731525332.pdf>
