# Supplementary material for: Magnetism in Nursing Education: A Qualitative Embedded Case Study of High‐Applicant Nursing Programs Amid a National Decline
Source: J Adv Nurs. 2025 Oct 28;82(6):6586–601. doi: 10.1111/jan.70295 (PMC13176678; doi:10.1111/jan.70295)
Supplement: Supplementary file 3 — Table S3: University cost of entrance examination and fees. [file JAN-82-6586-s001.docx]

**Supplementary Table 3**. University cost of entrance examination and fees

| **Bachelors in Nursing Science** | **Entrance examination cost (€) *** | **Fess, Maximum per year (€) *** |
| --- | --- | --- |
| U1 | 110 | 3,656.00 |
| U2 | 70 | 2,440.00 |
| U3 | 45 | 1,850.00 |
| U4 | 55 | 1,945.00 |

Legend. U, University, 1, the number; *source of data = webpages of the universities
