# Supplementary material for: Magnetism in Nursing Education: A Qualitative Embedded Case Study of High‐Applicant Nursing Programs Amid a National Decline
Source: J Adv Nurs. 2025 Oct 28;82(6):6586–601. doi: 10.1111/jan.70295 (PMC13176678; doi:10.1111/jan.70295)
Supplement: Supplementary file 4 — Table S4: The trail code. [file JAN-82-6586-s004.docx]

**Supplementary Table 4.** The trail code

| **Theme** | **The strategic location of the University** | |
| --- | --- | --- |
| **Subtheme** | **Codes** | **Quotations** |
| Accessibility | Proximity of the University to the place of living | “I immediately chose the U1 campus for purely logistical and geographical reasons (...) it was much more convenient in terms of public transportation than [another university] that was also close by, but the U1 was easier to get to because of the public transportation” (S)  “Compared to where I come from, where for example I have to travel a long distance to go to university ... here instead I have it just a stone's throw from home, so it was much easier, so maybe this is one of the main reasons, yes” (S)  “Yes, let's say that from an economic perspective maybe it was better to choose the closest one (...)” (S)  “...the main issue is related to the fact that we are in a place like U1 in [name of the city], that is actually not connected with the centre of [name of the city], but it is outside, so students who choose us do so either because of proximity, which means that they’re from this area” (CCP) |
|  | Transportation to the University and to the internship sites | "I immediately opted for the U3 , just for mere logistical and geographical reasons, (...) U3 location was much more convenient from the point of view of public transportation than perhaps the [another university] location, which is another location that is actually quite close to me, however U3, just because of public transportation, was easier to reach (...) to get there by public transportation…”(S)  “I took the entrance test and had been taken at another university, but it was ruled out because of a few elements, for example, the inconvenience of getting there, especially that” (S)  “The logistical aspects, we are on a university campus (...) however, it is easily accessible” (D)  "As for the logistical aspects, …This is especially true when we consider the internships, because if it's a matter of getting to class at 9 a.m. and stay until 6 p.m. then it's easier, but the working shift with the obviously different schedules makes it more complicated” (CCP)  “...At the logistical level there’s the reachability of the location and the possibility to carry out the internship according to what are the shifts of nurses and therefore having the possibility to do it even through their own means...” (CCP)  “Logistical aspects, it definitely makes a difference, the ability to move around, to have effective, efficient transportation, compatible with the course schedule, that definitely helps” (CCP)  “...I think maybe the location (...) it has a large parking area; it can be easily reached by transportation...” (CCP)  “...the proximity to the hospital facility...” (CCP)  "...... the logistical aspect is what I told you before, that is really the proximity between the hospital, so students know they’re attending at least 5-6 internships out of the total 8-9 there, with the university, which is why for example in [the name of the city] the situation is different in my opinion and the numbers are a bit lower...” (CCP) |
| Sustainability | Sustainability of the cost of living | “It’s absolutely not an expensive university, and life here (…) is very sustainable” (S)  “...the costs, even among the various options, since this is absolutely not an expensive university, and also the cost of living at least here in [the city] very affordable and sustainable...” (S)  “...the fact that being in [the city] the rent is very expensive, this is definitely not attractive, so let's say that even if you want to look for a house nearby, it's hard to find it, because they cost so much anyway, or are badly connected...” (S) |
|  | Support from relatives and acquaintances | "...I had enrolled in [the city] I'm talking about 2014 (...) I always considered the idea [of enrolling in Nursing], however, I always thought of moving to the North rather than to a university in the South (...) besides the fact that I had a support anyway, an aunt who lived in [the city] (...) so I knew I wouldn’t have been alone” (S)  “...mainly economic reasons, because I had two options anyway, either going to [another city] or coming here, because it was the only way, they were the only two places where I had people supporting me from the housing perspective...” (S)  “I have someone supporting me here since my father lives here, so I have his support, and I thought that this was the better option between those two alternatives” (S) |
|  | Housing availability | “We have residence halls very close to campus, and students can choose to live there or find other housing nearby” (S)  “...it is an area that is not so attractive geographically, because we are in the south of [the city] with a level of connections and hospitality for students that is not particularly efficient, so on the Campus about 5 years ago a student housing facility was also built, but even there the costs are quite high” (D)  “...we have housing facilities that are located right next to the campus, and students can choose to live there or find another facility nearby or come here just to attend classes and so on, but there is the possibility for those who are off-campus to live inside the campus as well, in the university facilities” (CCP)  “...there is a share of students, about a third, who come from outside areas, so this could be related maybe to the availability in terms of housing, of accommodations, for example” (CCP)  “...probably students could take into consideration the logistic aspects, without a shadow of a doubt, the possibility of finding a housing solution or something supporting their attendance, because (...) attendance for our program is mandatory, so it is essential to attend classes, you don’t have the opportunity to attend classes online and then go there in person only when you can...” (CCP) |
|  | Economic incentives | “The possibility of receiving scholarships or discounts on tuition fees (…) basically having financial advantages”(S)  “Other attractive aspects are the benefits granted by the region, such as discounts on university fees, because our students receive a whole range of benefits” (S)  “Also having opportunities regarding maybe scholarships or fee reductions (...) having benefits more than anything else, that's it” (S)  “Other aspects are related, for example, to the benefits that are granted, which depends on the region, for tax breaks concerning tuition fees, because our students can benefit from several subsidies” (D) |
| Geographical attractiveness | The appeal of the city, the university, the place in general | “Knowing that we were in a smaller centre gave them a sense of security, that we would be looked after more (...) we felt a bit more supported, not lost amongst the thousands of departments that you can find in larger universities” (S)  “...in my opinion [the city] is the best, generally speaking...it is definitely a protected environment, a small city, which translates into safety...” (D)  “The other locations are in the province of [the city] in a more central area that has always been quite attractive” (D)  “...one location in the area of [the city], in [the place], where we have another hospital, which is in a somewhat more difficult area, in fact even the places available are a bit less...” (D)  “...related so much to the university campus, which is small, because we are a small university, there are not so many courses, which is something that some people underlined (...) knowing that we are a smaller centre, which made them feel more confident that they would be taken care of in a better way (...) to feel a little bit more pampered because they are not dispersed in the myriad of departments that there can be in bigger centres.” (CCP)  “It still remains an average small university, which is also helpful to parents who support their choices, speaking of those graduating from high school and directly accessing the Nursing degree programme...” (NE)  “...the [the city] location attracts more people from outside the region (...) if people have to move from Southern Italy they choose Udine, maybe because it is more central and it is a little bit more known, it is a small town but still simple, and anyway we have a good university (...) while [the name of the city] attracts more people from the Veneto region, also in terms of logistic convenience...” (CCP)  “In part, I think there still exists a regional vocation of our course, that is, convenience, because anyway it is a fairly central place regionally and certainly, from a logistic point of view, this is useful” (CCP)  “...the university of [the city]…, I chose to come to [the city] because it was a bigger city anyway...” (S) |
|  | Willing to experience something new | “First and foremost, I wanted to move away... and immerse myself in a different environment to grow academically, professionally and personally (...) Life away from home played an important role, I needed personal growth, so I decided to leave without even considering my hometown” (S)  “...mainly wanting to go outside, right out of my comfort zone, trying to immerse myself in a different environment than my usual one, growing both academically, career-wise, work-wise, but also personally (...) the off-campus life also played a key role, I had the need to grow personally, so maybe I decided to leave and I didn't even take into account my own territory, I decided to really try to go outside...” (S)  “Personal reasons so I preferred to move to another place, to leave everything” (S)  “I had so much desire to gain experience, so I really wanted to move to a different city, not because... let's say that I had a desire to grow, because being 18 years old I really wanted to explore the world, so let's say that I didn’t even take into consideration the nearby locations, but simply because I didn’t want to stay home...” (S)  "...through the university experience I also wanted to embark on the experience of going live on my own, to gain independence from that perspective as well” (S) |
|  | Leisure and recreation | “They organize a lot of small parties, even sporting events, which really excite me. This gives me the feeling that university life is not just about studying, but also about enjoying and balancing everything” (S)  “When I first walked in, I literally got goosebumps; it says ‘This is a university for protagonists’ above the entrance: They make you feel welcome, valued and like you really belong” (S)  “There is a large tree-lined avenue, the football pitch, basketball and volleyball courts: There are regular tournaments organized... ...…there’s even a piano in the main hall, so the environment itself is really pleasant to live in” (S)  “...just for us students, maybe to bring us a little bit closer to this world, a lot of little parties are organized, even at the sports level, which really make me immerse myself in it, make me think maybe that university life is not only about studying all the time, but there is also something more and you can combine very well both the university part, let's say, and also the fun part, both things, finding the right balance” (S)  “...from a logistic point of view anyway you're in a big city, you're still in [the city], so a big city where as a young person you can definitely do a lot of things and it's very exciting, so let's say that this also led me to choose U3 over different locations” (S)  “Sometimes you also organize event where you watch movies together, for example, there are a lot of clubs, I know that there is the crochet club, for example, that meets every Thursday to watch a movie, to do ... so anyway ... I'm not aware of all these clubs or activities that there are in our university, however for sure for someone who also wants to be involved there are really a lot of things” (S) |
| **Theme** | **The reputation and influence of the nursing program** | |
| **Subtheme** | **Codes** | **Quotations** |
| Reputation | The ‘brand’ | “They choose [the city] both for the U1 brand (...)… then has a very long history in nursing, it really is a socially recognized profession, so [name of brand] is very interesting from that point of view” (D)  “in my opinion, is chosen precisely because of the type of the campus, and the U1 brand” (D)  “Maybe in a postgraduate career path seeing that you have a degree with the words ‘the name of the University’ makes you more important in a way” (S)  “I think the reputation of the university is one of the elements that leads the student to choose the course...” (CCP) |
|  | Ranking | “When I typed ‘best hospitals, best universities, nursing, Italy’ into Google, it was always a prominent name” (S)  "...I had enrolled in [the city] (...) I'm talking about 2014 (...) I always considered the idea [of enrolling in Nursing], however, I always thought of moving to the North rather than to a university in the South (...) Yes, I had done some research on the internet [on university rankings and scores]” (S)  “But searching for university rankings I saw that the University of U1 was ranked higher than the University of [name of the city], so in the end I still opted for the University of U1" (S)  “I thought it was more of a teaching-based thing [ranking], I don't know if I'm wrong...however, no, I didn't go into it” (S)  “...I had also researched a little bit about what was the position of the university in the national rankings, however the university of [name of the city] ranks second (...) so anyway I had taken that into consideration as well” (S)  “I definitely took into consideration the national ranking, I had looked at the CENSIS ranking itself” (S)  “...however, even national rankings or however in general by just typing in Google best hospitals, best universities, nursing, Italy, I mean, anyway it's always been a recurring name” (S)  “Yes, in my opinion, the CENSIS rankings, for example, but this works more, as I told you, outside the region, not so much at the local level” (NE) |
|  | Reputation/former students’ word of mouth | “More than any other official channel, talking to already enrolled students, near graduates and graduates was crucial for me” (S)  “I met a lot of fellow nurses who had attended the University of U1 anyway, both the younger ones and maybe the older ones who nevertheless spoke very highly of it and so ... there was also this reason, a little bit, because it was an excellent university anyway” (S)  “...it was actually the word of mouth; I relied a little bit on the people close to me because I actually have another university that’s like 10 minutes away from home...” (S)  “...more than feedback or maybe other official channels, even the website itself, for me the students already enrolled in the program and also undergraduates and graduates (...) in my opinion it's crucial to engage with students who are already enrolled because they can really give a fundamental contribution” (S)  “...I had heard about, I mean, I had talked to people who had studied health professions, so I had exchanged advice with them, I had asked for opinions (...) personal opinions of acquaintances” (S)  “...I took the entrance test at several Universities and was also accepted at others, but I chose this one because they spoke really high of it. I had two other friends who are now in their third year and who had already started their academic career in U 1 and they always spoke well of it, and told me that they were very comfortable with it, and so I decided to come here too” (S) |
|  | The modernity of the structures | “...as a structure, talking about the building itself, it's much newer, more structured (...) the other structure over here is a traditional old structure, with few rooms, small classrooms, [the name of the city] instead has in short quite big classrooms and it's new basically...” (S)  “...when they come to visit the facilities, the location, they can see the Simulation Centre, with the classrooms with digital whiteboards, as well as the platforms that are made available to the students, or simply the lockers, which is something a little bit... not innovative, but maybe resembling the American model, so they have this locker in the university where they can leave their things...” (CCP)  “...the facility is very new, it has been 10 years since it was born here physically with what we call the Campus, it is precisely in the same space where the hospital is, and the hospital facility is also relatively new, and visually it is beautiful as a structure, and very well maintained too, there are many green spaces, there are sports fields, so students can come out of the Campus and see the garden with benches (... ) there's a whole tree-lined area, there's fields for soccer, basketball, volleyball, tournaments are organized, (...) there's a piano in the lobby, we have study halls, the café, the study spaces, the study rooms, so it's just nice to experience the environment itself, so as for that I would say we're appealing, because even just the spaces where you experience the university environment are visually very nice, so that has a kind of impact” (CCP)  “...definitely this [the simulation center] is a strength of ours, which we also highlight a lot, meaning that this aspect always emerges, we also let them come to the simulation center to do these projects, sharing them with other professions as well, we show movies; it's also because of how we highlight the simulation center that we are able to attract students, or at least interested people, to help them understand the professions” (CCP) |
|  | Reputation of the hospital | “...the fact that I'm in a state-of-the-art hospital, which has also been awarded for many innovative activities and has always been one of the best hospitals in Italy anyway, according to the rankings, so I thought, since the university is anyway in collaboration with the hospital where I'm going to do internship then, it seemed like a good choice anyway” (S)  “...the reputation of the hospital in general, as I've always heard good things about it anyway, and even doing some research before I enrolled, I always found good information, or at least it inspired me, I had never seen anything particularly negative...” (S)  “...the reputation of the reference hospital because ours (...) is a hospital that has a very good reputation” (D)  “...word of mouth... certainly the name of the hospital that is also within the name of the [the name of the hospital] is still, despite some objective weaknesses that there are also in the hospital, it still helps from this point of view” (CCP)  “On the reputation of the hospital, yes, in my opinion it has an impact, and we match with the hospital, being part of the University group, we benefit a little bit from that, so I think it also influences maybe parents a little bit more, since maybe they know the facilities, more than students themselves” (CCP)  “Your previous experience in a hospital may give you an idea about [the quality of the hospital itself], but I don't think students consider the reputation of the hospital when enrolling. In any case, I don't think it's something they think to take into account” (NE) |
|  | Reputation of dean/professors | “...the thing that I took into consideration the most, precisely provided by this word of mouth, is related to teaching, that is, the reputation that the deans have, they spoke very highly of them to me (...) I was really recommended this kind of teaching, that is... the lecturers that are in my program now (...) mainly I had been spoken very highly of the lecturers (...) those people who manage a little bit the whole course...” (S)  “...I definitely considered the quality of the teaching, I came to know of quite experienced teachers, so I was definitely struck by that...” (S)  “Now that I've been able to experience for real what the Nursing program environment is here in [the name of the city] , definitely the reputation and the professionalism of the professors and the teaching mentors that are there within the University” (S)  “...there is an institutional figure, a disciplinary sector reference that in [the name of the city] was one of the first reasons, this is no small thing, that is, given a university role (...)in my opinion this has allowed to base this course on best practices (...) in my opinion it is fundamental because it has made it a program that has become a point of reference, because it is so, at the national level as a novelty and so on...” (D)  “it's not the reputation of the dean or the tutors that makes the student choose a specific university” (D) |
| Visibility | Nursing program promotion | “This is how students first learned about BNSc program activities and then decided to explore and participate” (S)  “...in the hospital there are several screens where the entry U1 University appeared, I saw some students, even from the Nursing program, so from there I immediately got the idea to go and do some research and see this University. And then right from the hospital anyway you can see it, because the campus is next to the hospital” (S)  “...I was invited by a local television station together with the professional association to talk about the degree program, to emphasize maybe the elements of excellence, in short...” (D)  “For another target of people you have to use other strategies, though, because more and more frequently we also have adult people who decide to start this profession, and so some activities are really focused on citizenship, not students, but on citizenship in general...” (CCP)  "We have been involved in a “third sector” activity: we participated in a festival that involved the whole city and held a workshop on hand washing for 4-5 year olds... when parents start to hear you talking knowledgeably about hand washing, they ask you, “What are you?”, 'We are nurses', so there is a need for dissemination activities of this kind. I believe that opportunities for dissemination at the third mission level should be exploited by the course of study in the hope of a positive impact in terms of attractiveness, but certainly to help spread a certain culture about the profession." (NE) |
|  | Digital-based | “...as for the university of U2, maybe I saw social media, to see what attracted me more anyway (...) when choosing the university of Udine, in addition to considering maybe the rankings, the scores of the universities, there was also the social media factor, so the videos, everything they had done, instead in 2014 I had only considered the scores of the universities and there was not so much” (S)  “...a lot of attention is paid to social media anyway, and in my opinion that's what makes students know this institution a little bit, anyway they have strategies with which they manage to attract a lot of students” (S)  “[Tutors] have the ability to pick up certain information through social media as well. Also, because then of course there are also emails, which is definitely the most used channel to communicate with students, however, social networks are also used” (S)  “Another thing, now we also do a lot of orientation through social media, which is definitely, for the target audience of high school students, the most effective, most immediate mode of communication, when you manage, however, to draw them into your social media (...) generally speaking, the word of mouth is definitely the best strategy, also to get them into social media, then when their attention is caught, captured through social media, this becomes a very good strategy then” (CCP) |
|  | Traditional based | “With activities where students can get hands-on experience to understand the different competencies that can be acquired” (S)  “[small group events] ...to attract those who would not have come spontaneously” (S)  “...trying to really get students involved, trying to get them to talk even during the open days, because I realized when I did the open days that students don't talk, maybe as a fifth grader I would have preferred to get more information from students, from people already enrolled in the program, for me the lecturer maybe talking and presenting the degree course wasn't that useful, but just having to deal with peers, or in any case even graduates talking to us a little bit about how they lived their experience at the degree program, what you can do next, what you can't do (... ) [bring out] the course of study though as seen through the eyes of the students...” (S)  “...I personally got these books where they listed the various degree programs, the curricula, however, then there was not that direct relationship with those who really deal with this university in a particular way” (S)  “And then after that I also participated in an online open day. (...) But now I'm sure they do them in-person as well, so it's another way to be able to get to people who maybe are interested (...) They definitely had given me more information about the entrance test, because there and then I didn't really understand what I had to do, what there was to do (...) Then yes, they explained to me a little bit what the educational offers were, which is already explained anyway on the website as well. More than anything else it was interesting to have been able to hear just the voice of some of these classroom tutors, who just explained a little bit about what this kind of experience is like, let's say” (S)  “My perception is that classroom interventions are more effective than student days or Campus visits. Let me give you a concrete example: we went to a fifth-year high school class and gave a lesson on first-year therapy administration, a lesson on heart physiology and first aid techniques, and practical exercises... this captures their attention much more and sparks their interest in a profession they may not have considered before. Now, I'll try to give you some data on effectiveness. Last year, I had five students, and one of them enrolled in nursing, so the effectiveness is 20%. If I compare that to this year's 17 students, 20% already becomes potentially three students” (NE) |
|  | The role of Champions:  Clinical nurses and students | “They know exactly how to focus on the positive aspects of the program, and they know the problems of their fellow students, so they know exactly what strings to pull to convince them” (S)  “Ultimately, applicants — and often their parents— - ask students questions, so they expect spontaneous answers rather than a formal presentation” (S)  “[nurses] explain who a nurse really is and what they can do” (S)  “There are also student-run sites that allow us to be even more immersed in that world … they give me a lot of reassurance; I really like what they show — simple things like the little parties they organize to make exam sessions less frustrating” (S)  "[Orientation activities] can involve students and the fact that we don't miss to involve recent graduates in these events.... meaning that our recent graduates, aside from the fact that we give them benefits, in the sense that we have the possibility, during the first six months, while they’re waiting maybe to start their job, to do practical trainings as well, some things, at the simulation center, that are organized annually... And in addition, some of them then even once they are done with their academic career, anyway they stay with us and do something, they support us for example with dissemination or even for other things...” (D)  “...we’ve been trying, in the last few years, to keep them involved...it's becoming easier maybe to keep former students involved, and in that sense, I could give you a list of at least ten students who have just graduated and have told me ‘contact me if there are things to do’ (...) certainly, in terms of cost-effectiveness as well, involving former students who have already gone through certain experiences turns out to be much easier [than involving clinical nurses] and indirectly a way to involve people who work in practice” (CCP)  "...I'm talking about nursing, but in my opinion, all [healthcare] professions need to make themselves known, because one of the questions students ask is “so what do you actually do?”, and while this is easier for a physiotherapist or radiology technician, it's difficult for us to summarise (...), but if you have practical activities, you can help them understand what it means and you can entice them with the idea of learning and understanding something that can also have an impact on their everyday lives" (NE) |
| Development | Promotion of career opportunities | “To also invest in what comes after graduation” (S)  “The role of the nurse is poorly understood in terms of prospects, opportunities, development and career” (S)  “There is a lack of information about educational opportunities, career goals and the potential of the profession” (D)  “...we try to illustrate what the postgraduate possibilities are, that is, so work-wise, but also study-wise, even a possible academic career, through for example all the steps provided, so bachelor's degree, master's degree, PhD, and we focus a lot on the fact that it is one of the most flexible professions (...) so try to make them understand that nurses also make careers, that is, in the most traditional sense of the word” (D)  "...the activities that we bring forward generally also start from enhancing the figure of the nurse as a competent professional who has his own scope of application, work and responsibility accordingly and who then also has the opportunity to make a career in this sense, so that he does not just stay fixed and stable in a certain situation, but that he has different possibilities, that could indeed be even much broader than those that other professions could have in the same field. So this is kind of the starting point, which is the fact that we enhance the figure of the nurse within the activities that we propose” (CCP)  “...a lack of information with respect to the educational offers, the professional outlets, the potential of a profession...if you don't know, if you don't know, if nobody advertises, obviously you are not inclined to choose that path” (CCP)  “The fact that at the societal level the figure of the nurse is poorly known in terms of even prospects, opportunities, development, career (...) even when talking to other university colleagues about this prospect, as well, I wouldn't be surprised if someone said - oh, so nurses can also be doctoral students?” That’s it, we go through this kind of experiences in different contexts, so the choice would be to at least try with those bodies that maybe have a little more appeal in terms of profession is a strategy that may have positive consequences in terms of ‘Oh, this prospect interests me, let's try to pursue it” (CCP)  “[Hindering factors] (...) could be maybe not finding an answer with respect to what one's career idea might be, that is, idea as a nurse in the future...” (CCP)  “The role of the nurse is poorly understood in terms of prospects, opportunities, development and career” (NE) |
|  | Availability of a master’s degree within the same university | “I think the other thing that's promoting attractiveness anyway is also another kind of message that we have started to share since 2020, which was the development of the clinical master's degree (...) as soon as the new regulations on nursing master's degrees are enacted, we'll be ready to start with at least two specializations courses, at least two, so I think that has an impact as well” (D)  “...the possibility of having a master's degree program in the same location, for example, maybe even with a new perspective, now in [name of the city] one of the first master's degree programs in nursing and midwifery science with a more clinical perspective has started, so more related to the family nurse, for example, which might be an attractive element for those who maybe know that they like to study and would like to complete their five-year educational path” (CCP)  “Offers a Master’s degree within the same institution” (NE) |
|  | Job prospects | “We don't have to go through the whole recruitment process: If we are found goo students, we can start working after just one interview” (D)  “...retention rate is 100 percent, that is, when you graduate you are sure that if you want to work at [the name of the hospital] you will get in immediately, so you get different types of advantages in terms of hiring prospects when compared to the public health sector” (D)  “Someone over time (...) chose the Bachelor’s Degree in Nursing because of the job opportunities after graduation” (CCP) |
| **Theme** | **A Structured, Innovative, and Open-Oriented Nursing Curriculum** | |
| A well-designed curriculum | The sequence of activities respects the learning process | “We prepare them in an initial theoretical phase before they go into the clinic, always through simulations, exercises, clinical cases and clinical reasoning. This intermediate phase reassures and prepares them (…) they feel more confident and less anxious” (S)  “The coherence of the parallel treatment of topics, lectures and exercises facilitates learning and helps to better memorize the concepts” (D)  “I completed a critical care practicum in December and took the exam in January, applying the concepts I had practiced in theory and practice” (D)  “a completely different didactic system, not made... not divided into lectures, then sessions and then internships, let's say the fact that it's spread out, not divided into sections, but having six exam rounds, rather than one round, two, only in a given session, let's say that this was an advantage” (S)  "...unlike other people, it's positive because I can spread my exams out over the whole year, I can manage them, I have six possibilities, six rounds for each exam, I can decide whether to take one round or the other, in my opinion it's much better than managing it like other universities do with sessions, because you have that, just the exams, all the exams together, while spreading them out over the whole year in my opinion is a very positive thing, much more effective (... ) Because you have so many possibilities spread over the whole year, and you decide when you want to take them -- when you really want to take that exam, and you manage your own time” (S)  “I had been particularly impressed by the educational offer that the course ensured, (...) especially by the alternation between theoretical lectures, practical classes, then laboratory classes, and actual internships” (S)  “U1 is of very high quality, both from an educational perspective, which is one of the elements that we gather a lot from the students, that is, they know that if they come study at U1 in [the name of the city] it is because we have stated that they’ll get a standardized training model like the one in [the name if the city] , and there the educational quality in some areas is a bit low, so they are very attracted to that, in addition to the advantage of finding a job immediately after that” (D) |
|  | The degree of integration facilitates a meaningful learning | “If you do a morning shift and have lectures in the afternoon, you can't fully engage with the shift and approach the lectures properly” (S)  “Having six exam dates spread throughout the year, where I can schedule my exams independently rather than having my time managed by the university… You know you have to concentrate on theory and have no other worries” (S)  “...the fact that they delve into some subjects, some modules compared to perhaps other degree programs where they are not delved into maybe” (S)  “The fact that we use the Teams channel (...) having a Teams channel where the lecturer adds insights, articles, their teaching materials, it helps so much to support us with our study process, but also to point us to what we need to study (...) we find everything on the Teams channel, and it is an advantage for us” (S)  "... with the lecturers however you have a relationship, let's say, that is face to face, it's not a lecturer, maybe a classic university lecturer teaching in a classroom with 300-400 people, who maybe is difficult to reach, does not respond to emails, even distant, because anyway they have a much larger number of students, our classes instead are not that crowded and we have the lecturer just in front of us who talks to us, involves us, if we need or we can find him in the corridors, after lectures, or even when we send emails they respond promptly, all these good things in short”(S)  "...it's interesting, the lectures, even the somewhat heavy lectures are organized in a more interactive way. Definitely that whole aspect, precisely of the lecturers with frontal lectures, the interactive classrooms...” (S)  “...it could also be the educational offer of our didactics, in any case the program involves professors of depth, also important ones (...) for the quality of the program, of course one has to be convinced to do nursing, that's the sine qua non” (CCP) |
| A tension towards innovation | The teaching methods are innovative | “The lecturer invited some students to give a lesson; for me that was a wonderful experience (…) I realized how challenging it really is, and it was also very, very satisfying” (S)  “...the aspect that comes out the most when talking to students, they are interested in the innovative teaching methodologies, the spaces, the campus that also proposes the possibility of being in the same space with international students, still being a very young, innovative context, that even just looking at the university life, they see it as something different from other older venues...what they tell us is just that, at the level of attractiveness, it's also related to the innovation that we propose, they mainly see this element as appealing” (CCP)  “...we benefit from our image of a young and innovative university...” (CCP)  “...the university impressed me more because it was more innovative” (S)  “We are a location that has four courses of study in nursing, one here in [the name of the city] on the University, which is practically attached to the main hospital of the U3 group in [the city], and this is a strength on the one hand, because it is a brand that attracts in terms of specificity, technology, high specialization...” (D) |
|  | Innovative clinical placements | “Lots of contexts in which to gain experience - not just the hospital, but all other environments, including outside (…) seeing the reality of other hospitals, large and small institutions...” (S)  "We have also taken into account how the needs of the population have evolved and in response we have included these types of experiences in our training package” (S)  “Many contexts in which to gain experience, not only the hospital but all other environments, even external ones (…) seeing the reality of other hospitals, of large and small institutions…” (D)  “Also considering how population needs have evolved, so in response to this, including these types of experiences within our training package” (CCP) |
|  | Advanced laboratory opportunities | “Having labs where to challenge yourself, so the simulation center, which is something I had never heard of in any other contexts, so having state-of-the-art tools with which we can practice before we take on the challenge of the internship” (S)  “...related to teaching, in my opinion workshops are a very attractive element, that is, the possibility to experiment a little more during the lectures period...” (S)  “On the methodologies that are used as well, so workshops, the aspect of interprofessional seminars...” (CCP) |
| Networking | Immersion in a professional and social environment | "...having however an international university and it is also connected with the faculty of medicine, there is the possibility to interact also with the other degree program (...) through societies that exist within the university, so for example there is the society of Gynecology, Pediatrics, Neurology, so a little bit divided into clinical specialties, so everyone according to their interest, (... ) for example we're going to attend in two weeks a course with the American Emergency Surgery Association and it's open to all undergraduate courses, and I think this is a good activity to be able to interact with all the students, expand their knowledge and create, develop a network, and why not, even being able to practise your English a little bit by being with international students anyway, with a good interaction” (S)  "when they take the test they also state the location they want to choose, after that, on the basis of places, they are very flexible, for example students who maybe entered the ranking and had chosen [the name of the city] , but then [the name of the city] got immediately saturated, they attended for some time either the context of [the name of the city] or the one of [the name of the city] according to their needs, possibilities, and then we tried to move them as quickly as possible by having them return to [the name of the city], and vice versa, we had students who had enrolled in [the name of the city], then found their partner in Lombardy and asked to be moved, so we are very flexible from this point of view” (D)  “it's a great opportunity to be part of the national conference of the degree programs because we always compare ourselves with what emerges from the conference (...) it's very useful, it's a very valuable opportunity, we compare and take ideas, we keep working, so it's really useful” (D)  “I must say that both with the companies, the main one, the health care company, there is really a common purpose, they always give us very good indications on the preparation of our students” (D)  “...there's a very good collaboration with the Board of the Nurses (OPI), so students who will graduate in the future, the OPI organizes an extraordinary session to provide them with the number and the qualification for the profession...” (CCP)  "And also, with the Order of the Professions (...) for example I was mentioning that tomorrow night there will be this broadcast concerning nurses and the president just asked me if I could also speak about the degree program, in addition to those activities that we organize in the schools, where they also come, those days involving our citizens. So yes, we are very pleased with this collaboration because I think that the degree program alone cannot do everything, there has to be a unity of spirit within the community, in the society that is working towards the same direction” (D) |
|  | International perspectives and experiences | “With internationalization in mind, a project called Honor Track was launched last year. It offers students the opportunity to do volunteer work and internships abroad, especially in countries with few resources. The proposed destinations last year were Madagascar and Tanzania. I applied, received the scholarship and was able to gain a valuable educational experience abroad” (S)  "...the international relations of our university, I mean of our degree program, so in a nutshell the opportunities to study abroad offers, so the Erasmus proposals. Because there are a lot of destinations anyway and they are very, let's say, scattered, a little bit all over Europe...” (S)  "...I mentioned earlier the possibility of international mobility that I found out that U1 offers because before I enrolled, yes, I had read it in the student guide, but I didn't really understand what they meant. [the name of the city] offers the possibility, for students from the second year, in addition to participating in Erasmus, to participate in projects that the university has thought of, so volunteering (...) or offering a travel grant, based on a project that the student chooses, all over the world, so the university allocates a fund to cover or partially support the student's project (... ) I think this is a great opportunity since there are students who have used it and the university is always there to encourage students to also take advantage of this opportunity, as it certainly enriches the student personally anyway, and then it could also benefit the university, meaning that anyway these experiences at the beginning of each academic year are then shared with all the other students, with new students and anyway with fellow students. (...) in addition of course to all the possibilities of Erasmus or international projects of the university” (S)  “...expand the possibilities for students not only in the contexts within our hospitals, but also by proposing activities that have a somewhat more international scope as well, so trying to develop collaborations and cooperation with foreign universities both for short exchanges but also for longer exchanges such as the Erasmus experience, which is the most traditional project that we can be involved into” (CCP)  “The possibility also to go abroad, we focus a lot on internationalization, both with internship paths abroad, but also with perhaps shorter, shorter activities, which still allow a direct interaction of students with other students from different countries and on different topics, which can be clinical, care-related, but also organizational, and also with other professions, because sometimes these projects we developed with other universities are multiprofessional” (CCP) |
| **Theme** | **The quality of the tutorial system** | |
| Being welcomed | Being included | “Another thing is (...) I don't know if it's a matter of luck or if it's... is that there was a nice bonding in the classroom with everybody from the very beginning, so yes, it was an inclusive approach” (S)  “As for me, during the first days I was in the university all day because I found it was beautiful, but just because they make you feel welcomed, you come and just feel welcomed” (S) |
| Being always supported | Accessible nurse educators | “...students are very well looked after because there is this network of nursing teaching staff who are very helpful, who are always at the college and who also respond immediately to emails, even if there are problems with lecturers or professors” (S)  “That was one of the aspects that impressed me the most: the fact that you are followed and not left alone, that you are supported” (S)  “There is no real distance between student and the tutors: you feel part of the course” (S)  “One-on-one meetings are held once a semester between each student and their tutor to identify strengths and weaknesses” (S)  “...also the nurse educator to refer to with respect to the theoretical part instead...” (S)  “Students are very well taken care of because there is this network of nurse educators who are very helpful, always at the university, who also respond immediately to emails, even if there are issue with lecturers or professors do not respond directly to our emails, educators act as a go-between to get a response more quickly” (S)  “...the opportunity to be able to always count on our reference supervisors, who are available for any needs...” (S) |
|  | Having clinical supervisors | “The opportunity to help understand how to navigate a unit is a very attractive feature”in my opinion” (S)  “...yes, related to the internship also, the fact that you have supervisors who accompany you during the whole internship period, who are next to you, who support you, who teach you, who don't leave you alone anyway, at the mercy of the department” (S)  “the tutorial system in the first place (...) talking to my cousin (...) they don't have any tutorial system during their internship, they are just catapulted in the different wards and (...) they are joined by some nurses, different ones, day by day, but there is no linearity, or continuity over time, today we’ll learn this, tomorrow we’ll learn that (...) that’s what happens” (S)  "Here you don't, that is, you have your supervisor, you define what goals you want to achieve together with your supervisor, what you feel you are better at, what you feel you are less good at, that you need to deepen, and the supervisor is investing his time, all his skills, investing on you. And you rely on him anyway, because you know you can trust him, you know he can guide you in achieving your goals...” (S) |
|  | Being supported by an integrated tutorial system | “It strengthens the integration between the clinical and theoretical components” (S)  “The tutorial system, both clinical supervisors and nurse educators, during internships and also during classes, so lectures, exams (...) the fact that you really have a support network” (S)  “...there is a lot of synergy within the internship locations and companies, so the internship supervisors often meet with the RIDs (Department Nursing Coordinator), the nurses, we just try to work together” (D)  "...combined with the tutorial system that we are able to build, both in the clinical setting, so both with the internship supervisors, but also probably with the tutorial system based on full-time nurse educators. There's a lot of work done from that point of view” (CCP) |
|  | Giving and receiving peer mentoring | A very useful thing (...) during the first-year internship, during the first two weeks you are supervised not only by your clinical supervisor but also by a third-year student, and this, at least for me, was helpful in decreasing some of the anxiety of the first internship, because you were also interacting with another student, so it was more of a peer-to-peer approach than with the clinical supervisor” (S)  “Another attractive thing is the labs, again from the first year, some of which are delivered by third-year students (...) so there is less fear of asking questions, you feel a little less ‘judged” (S)  “...the first few days of university, maybe even during the first day [the mentor students] came to our class to introduce themselves, so then they kind of explained to us what their role was and so on, so definitely if I had any doubts and I saw them around...I mean I had asked them something, definitely even a little bit… not from a practical point of view, but something more like “oh god, it’s my first days at university and I don't know what...what I should do, how I should organize my exam session, whether I should take an oral exam, please give me some advice, what should I do” (S) |
| **Theme** | **The program’s commitment to student support and development** | |
| Being valued as a student | Active student participation | “We involve them heavily, discuss with them, analyze the comments on quality, the data collected, it's a team effort” (S)  “...I collaborated with the lecturers to get new simulation activities going, so we are given a lot of opportunities to develop new ideas with the tutors...” (S)  "...how represented and active our students are in the various university bodies, I can think of the council, the CAQ [quality assurance committee], a teaching committee. They are very active, all the course years are represented, they are usually doubled, that is we have two students per year and that too, in a nursing CAQ we have three students, and we also have a recent graduate, who’s the one who keeps track of older events, which is very interesting, and we also have a good representation of faculty, we have a RAFP (Coordinator of the professionalizing teaching activities), so it's a well-structured group... as for me, I think the CAQ is the most interesting thing we have in terms of councils (... ) The students, something that is lacking in other courses, at least in my experience, there’s a high number of them and this naturally creates a virtuous circle, because clearly I mean, you can have a real impact on a student , then if students are smart, as they are, from there you can take inspiration to... because you know when we talk to each other, you also struggle to understand where the problems are. Or your problems are not problems and you don't perceive the problems of the students” (D)  "...I always try to involve them [the students] very much by also organizing focus groups, discussing with them the comments about the quality, for example, the evaluations they report to me about the internship, the lectures and so on, what I have gathered from them and what they like very much is the important presence of the tutorial network, they need relevant human relationships (... ) I don't have a very large numbers [of tutors] but we manage to do a lot of in-person work by meeting them often, and we also work together with the internship guides, together with the hospital, so they feel very cared for, very welcomed...” (D)  “These students who work in the governing bodies, we also keep them involved with respect to the orientation activities, and so they become positive spokespersons, because even in relation to the possible critical issues that they point out to us, in terms of organizational improvement, training or whatever, they then become spokespersons in relation to what has improved, so when they go out to talk about the program, we have heard them state that what they really like is the fact that we welcome as much as possible their requests for changes or reporting of problems and we try to solve them by working with them, so this welcoming attitude, this alliance, yes” (D)  “...valuing them, valuing them on anything from peer mentoring projects to, I don't know, inauguration projects for the academic year (...) in March we managed to create a group of about forty students and we are taking them to the FNOPI Congress, they team up, we value them (...) these are events that we try to get them to participate in, to understand what kind of world they are going into, that they are not alone” (D)  “...a series of questionnaires that we use on different levels, so the didactics questionnaire, but also the questionnaires that we ask students to fill after their internship experiences, where they actually provide an evaluation on how the internship experience was, on the internship assistants, on the context that welcomed them, so these are all data that we subsequently reanalyse, go back to them to understand how to bring changes throughout the system as well, as a degree program as a whole...” (CCP)  "In the governing bodies, given that we have them, they have helped us probably because it was during one of our activities, if I'm not mistaken, the Joint Teachers-Students Committee originated from them, since we were talking about the problem of attractiveness and numbers, the proposal to receive recognition in terms of credits, so they have been very important in this because then they do it probably regardless of credits, but credits are a motivating element (... ) I feel that those who participate in the other activities such as the degree program Council or the Review Group, or the Quality Assurance Group, we see them very determined also when participating in these orientation activities” (CCP) |
| Being considered in the preferences and needs | Tailored educational pathway | “We are actually asked which departments we would like to do placements in… we have the opportunity to do placements in the areas we choose” (S)  “Students really appreciate that their wishes are taken on board” (S)  “can help precisely to improve, adapt the educational proposal that is addressed as well, which clearly does not apply to them anymore, because they maybe will no longer be part of the program, but when we listen to the others, maybe second-year students or first-year students who say “ah gosh, well, if this changes like this next year, it will impact us”so the development of a joint path, as far as possible...” (CCP)  “a strength is that during the second and third year they ask you which your preferences are, so you can influence your path, they are not always accepted, however they ask you about them” (S)  "...the interesting part, especially in relation to the third year is that, of course with respect to students who have duly passed their exams, you have the opportunity to choose, together with the tutor, the path that interests the student the most (...) in these three clinical experiences during the third year we are truly asked in which departments we would like to attend internship activities, and this is gives us great freedom as motivated students because we are actually given the opportunity to be able to attend internships being aware of the fact that it’s really us who are going to go there and choose what’s best. I see this as a strength of the university as well...” (S)  "...my curiosity to maybe attend an extra internship period, always within the hospital, out of personal curiosity, has been approved, so here again the opportunity of spending more time in the hospital (...) also with regards to the thesis, I am developing a research thesis and the university has really created a specific course for us students who are developing an experimental thesis. So even this whole part of supporting personal student projects or ideas, I feel that here I've been supported and not maybe limited or excluded” (S)  “that the teaching tutors ask for our opinion on how the academic year is structured, so last year we had a meeting, and they asked us for advice on when to schedule exams and labs... for example, not to include too many exams during the internship period, because it is a very busy and intense period, and find a solution in that sense” (S)  “there are some of my classmates who are working, so maybe for the organization of even the internships and their work they can ask them how to best combine everything (...) if they have any problems they go to them and ask for information about their work, to schedule the internship shifts earlier (...) these are little things that help anyway” (S)  “...from the third year we can choose the internships that we can attend and so it's very...it's also very nice to be able to kind of decide where to go and what we want to do a little bit with our path (...) based on how you are, let’s say (...) if you're on track with all the exams and you still have a good average they listen to you on with respect to all three internships, however it wasn't like that for everybody in our class here, however you can definitely choose at least 1-2 internships” (S)  “The other element concerns working students, that we have already mentioned, so trying to work on fostering pathways for working students” (CCP) |
|  | Study-life balance | "I manage to balance well both my life, maybe my social commitments, my personal life with my university life, I don't feel I have to give up some things because during that particular period I have that exam session, so like “this month I have to give up this in order to do this, this and this, I can’t go on holiday or go out because I have to lock myself at home to study for my exam session”, for example. Instead, I think I can do it, having six exam rounds spread throughout the year, so during March I’ll focus on taking a certain exam, then maybe in April ... well, even in March I can focus on that and take two exams, it depends on how I manage my time” (S)  “...just for us students, maybe to bring us a little bit closer to this world, a lot of little parties are organized, even at the sports level, which really make me immerse myself in it, make me think maybe that university life is not only about studying all the time, but there is also something more and you can combine very well both the university part, let's say, and also the fun part, both things, finding the right balance” (S)  “Sometimes you also organize event where you watch movies together, for example, there are a lot of clubs, I know that there is the crochet club, for example, that meets every Thursday to watch a movie, to do ... so anyway ... I'm not aware of all these clubs or activities that there are in our university, however for sure for someone who also wants to be involved there are really a lot of things” (S) |

Legend: BNS, Bachelor of Nursing Science; CAQ, Quality Assurance Commission; CCP, Clinical Coordinator of the Practice; CENSIS, Centro Studi Investimenti Sociali (=Who provides the annual ranking of the degrees); D, Dean; FNOPI, Federazione Nazionale Ordini Professioni Infermieristiche (=The National Board of Nurses in Italy); NE, nurse educator; U1-2-3, University 1-2-3.
